# Targeting FMN, TPP, SAM-I, and glmS Riboswitches with Chimeric Antisense Oligonucleotides for Completely Rational Antibacterial Drug Development

**DOI:** 10.3390/antibiotics12111607

**Published:** 2023-11-08

**Authors:** Nikolet Pavlova, Martina Traykovska, Robert Penchovsky

**Affiliations:** Laboratory of Synthetic Biology and Bioinformatics, Faculty of Biology, Sofia University “St. Kliment Ohridski”, 8 Dragan Tzankov Blvd., 1164 Sofia, Bulgaria

**Keywords:** antisense oligonucleotides, cell-penetrating peptides, drug targets, antibacterial agents, riboswitches, antibacterial drug discovery, rational drug development

## Abstract

Antimicrobial drug resistance has emerged as a significant challenge in contemporary medicine due to the proliferation of numerous bacterial strains resistant to all existing antibiotics. Meanwhile, riboswitches have emerged as promising targets for discovering antibacterial drugs. Riboswitches are regulatory elements in certain bacterial mRNAs that can bind to specific molecules and control gene expression via transcriptional termination, prevention of translation, or mRNA destabilization. By targeting riboswitches, we aim to develop innovative strategies to combat antibiotic-resistant bacteria and enhance the efficacy of antibacterial treatments. This convergence of challenges and opportunities underscores the ongoing quest to revolutionize medical approaches against evolving bacterial threats. For the first time, this innovative review describes the rational design and applications of chimeric antisense oligonucleotides as antibacterial agents targeting four riboswitches selected based on genome-wide bioinformatic analyses. The antisense oligonucleotides are coupled with the cell-penetrating oligopeptide pVEC, which penetrates Gram-positive and Gram-negative bacteria and specifically targets glmS, FMN, TPP, and SAM-I riboswitches in *Staphylococcus aureus*, *Listeria monocytogenes*, and *Escherichia coli*. The average antibiotic dosage of antisense oligonucleotides that inhibits 80% of bacterial growth is around 700 nM (4.5 μg/mL). Antisense oligonucleotides do not exhibit toxicity in human cell lines at this concentration. The results demonstrate that these riboswitches are suitable targets for antibacterial drug development using antisense oligonucleotide technology. The approach is fully rational because selecting suitable riboswitch targets and designing ASOs that target them are based on predefined criteria. The approach can be used to develop narrow or broad-spectrum antibiotics against multidrug-resistant bacterial strains for a short time. The approach is easily adaptive to new resistance using targeting NGS technology.

## 1. Introduction

Antibiotic resistance (AR), declared by the World Health Organization (WHO) as one of the top ten global health threats facing humanity, is a critical global problem that affects people all over the globe [1,2]. As a result of the ineffectiveness of antibiotic therapies, treatments in home and hospital environments are prolonged, the costs for medicines are increased, and even the mortality due to infections is increased. Different reports show that AR causes multidrug-resistant organism (MDRO) sepsis [3,4,5]. The global burden assessed across different pathogen-drug combinations in 2019 was an estimated 4.95 million deaths, from which one child dies every 3 minutes and 1.27 million deaths (children and adults) yearly are directly bound to AR [6]. A challenge for scientists in medicine and pharmacy is the discovery of novel antibiotics against which bacteria have not shown insensitivity until now. Basic research is aimed at the discovery of new targets, as well as the development of new therapeutic candidates that have a bacteriostatic or bactericidal effect. Over the past two decades, various targets have been tested and shown high suitability during their bioinformatics study and in vitro and in vivo experiments [7,8,9,10]. One of the most encouraging results is with gene control elements known as riboswitches found in the bacterial genome about 20 years ago.

Most of the 55 different known classes of riboswitches are spread in bacteria [11,12]. Only one class of riboswitches has been found in several plants and fungi. It is known as the thiamine pyrophosphate (TPP) riboswitch and regulates gene expression via alternative splicing [13,14]. Recent research has shown that riboswitches may be promising novels targets for antibacterial drug discovery [12,15,16]. Bacterial riboswitches are mostly located in the untranslated regions of different mRNAs. They perform a regulatory function by binding specific metabolites that cause changes in the production of certain mRNAs.

Our approach is fully rational, including two separate stages: target evaluation and drug design. We came to analyze the different classes of riboswitches, suggesting a system for a rational approach with clearly postulated criteria. As a result, we can precisely classify any riboswitch of a particular bacterium into four groups to develop new antibacterial agents [13,16]. After their classification, the most suitable riboswitches, for the first time, serve as targets in the subsequent rational design of antisense oligonucleotides (ASOs). We have proven that this fully rational approach has high efficiency, with four successful designs out of four [14,17,18,19], which can create broad-spectrum or narrow-spectrum antibacterial agents. The method is universal since it can be applied to any mRNA, including those without riboswitches.

ASOs are short single-stranded (ss) RNA or DNA molecules with an optimal length of 13–25 nucleotides that can directly bind to genome structures, such as mRNAs, bacterial riboswitches, or other nucleotide sequences, via sequence-specific hybridization and induce cleavage of its structure [20,21]. As a result of enzymatic cleavage via RNase H, the expression of proteins with an important effect on the survival and division of the bacterium is inhibited. In this way, ASOs elegantly regulate gene expression. To ensure the precise insertion of the potential therapeutic agent into the bacterial cell, a cell-penetrating peptide, such as pVEC or others, can be attached to the ASO’s structure [22,23,24,25,26,27]. Their specific design, rapid and directed targeting, and cell-penetrating peptide (CPP) make ASOs one of the most promising bacterial chemical agents.

## 2. Bacterial Riboswitches

### 2.1. Structure and Function of Riboswitches

The discovery of the first ribozymes added the biosensing function to natural RNAs’ diverse functional properties. Riboswitches are regulatory elements typically located in the 5′-untranslated region (5′-UTR) of different messenger(m) RNAs, which sense small-molecule metabolite concentrations for regulation of gene expression [7,8,28,29,30,31]. Their structure includes an aptamer domain and expression platform. The aptamer domain is a metabolite-sensitive structure typically in a length of 35 to 200 nucleotides, usually located in the 5′-UTR of mRNA with a highly conserved sequence. The aptamer forms a three-dimensional (3D) structure able to bind particular cellular metabolites, and it is used to classify different riboswitches. There are more than 26 ligands, such as amino acids, coenzymes, ions, nucleotide derivatives, and signaling molecules. Based on well-studied and -established conservative aptamer sequences, riboswitches are classified into 55 classes [11]. One ligand can be sensed in bacteria by several riboswitch classes, each with a different aptamer, such as, SAM-I, II, III, and IV riboswitches. Some SAM riboswitches have completely different aptamers, which implies various evolutionary origins and pathways.

**Figure 1:**
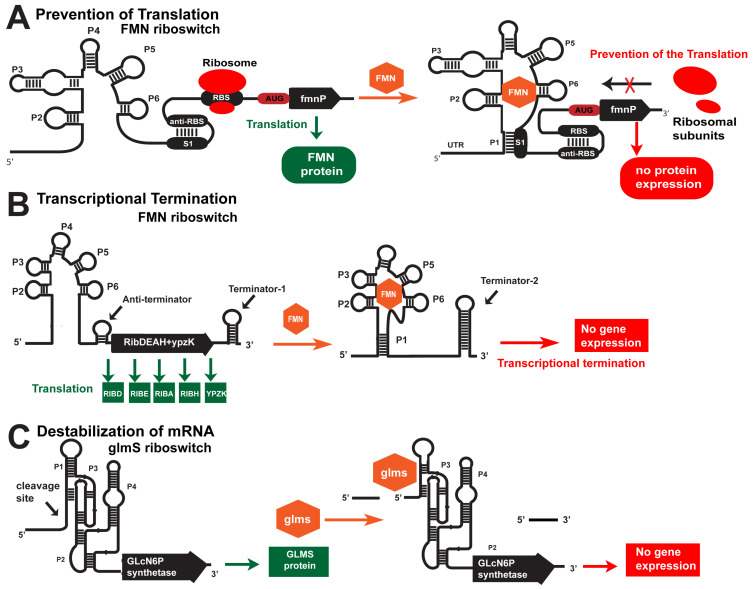
Cis-acting mechanisms of gene regulation by riboswitches. (**A**) Prevention of translation. In the absence of FMN, the RBS is accessible, and the small ribosomal subunit binds to mRNA. When FMN is present in the cell, it binds to the aptamer domain, which leads to a conformational change and hybridization of the RBS. As a result, the RBS is unavailable for the small ribosomal subunit binding. (**B**) Termination of transcription. The FMN riboswitch is located in the 5′-UTR of polycistronic mRNA, which encodes five proteins responsible for the biosynthesis of FMN in many bacteria. In the absence of FMN, the aptamer folds into a structure that allows for the formation of an anti-terminator and does not allow for the formation of a terminator near the 5′-UTR. As a result, the polycistronic mRNA is transcribed and translated into five proteins, including RIBD, RIBE, RIBA, RIBH, and YPZK. When FMN is present in the bacterium, the aptamer folds into a structure that facilitates the formation of terminator-2 in the 5′-UTR. As a result, the transcription of the polycistronic mRNA is prematurely terminated. (**C**) Destabilization of mRNA. The glmS riboswitch is a unique metabolite-sensitive ribozyme found in the 5′-UTR of mRNA encoding the enzyme glutamine fructose-6-phosphate aminotransferase. In the absence of glucosamine-6-phosphate (glmS), the ribozyme is inactive, and the glutamine-fructose-6-phosphate aminotransferase is expressed. In the presence of glmS, the ribozyme self-cleaves its structure by destabilizing the glmS mRNA.

There are three cis-acting mechanisms for controlling gene expression by bacterial riboswitches, such as preventing translation (Figure 1A), transcriptional termination (Figure 1B), and mRNA destabilization (Figure 1A,C). The first two mechanisms are widely spread, while mRNA destabilization is restricted to the glmS riboswitch. For instance, the prevention of translation occurs with the fmnP gene in the presence of high FMN concentration in the bacteria. FMN binds to its aptamer in this case and forms P1 via hybridization with the S1 sequence (Figure 1A). As a result, the anti-RBS sequence is free to bind with the RBS sequence, blocking the translation of the fmnP mRNA. In contrast, when FMN concentration is low, the stem P1 is not formed, and the S1 sequence hybridizes with the anti-RBS, which sets the RBS free for initialization of translation (Figure 1A). As a result, the FMN transporter protein is expressed. The termination of transcription is depicted in the ribD operon that encodes all enzymes for the FMN synthesis. Again, the same FMN aptamer senses the concentration of FMN. In a low FMN concentration, stem P1 is not formed, but a small anti-terminator stem is formed that allows for transcription of the whole polycistronic mRNA (Figure 1B). Conversely, stem P1 and terminator-2 are formed within the 5′-UTR in a high FMN concentration (Figure 1B). That terminates the transcription, and only 5′-UTR is synthesized. As a result, FMN is not produced. The third mechanism is mRNA destabilization via metabolite (glmS)-inducible self-cleavage ribozyme. In a high concentration of glmS, the ribozyme cleaves itself (Figure 1C). That reduces the half-life of the mRNA for the glmS synthetase, and glmS is not produced. These are the first discovered metabolite-inducible ribozymes that regulate gene expression in which glmS serves as a cofactor. Note that the riboswitches are kinetically driven senses, where the speed of the ligand binding to the aptamer domain is the main trigger of the chain of events altering gene expression in the OFF or ON direction. By targeting riboswitches with various ASOs, we destabilize the targeted mRNAs and block the synthesis of essential metabolites, without which the bacteria cannot divide or survive.

One riboswitch class can regulate one or more distinct metabolic biochemical pathways using the same aptamer domain. The expression platform of the riboswitch encodes between one and several proteins, such as enzymes or transporters. For instance, the FMN riboswitch can sense the concentration of FMN and regulate the expression of the fmnP gene, which encodes an FMN transporter, via prevention of translation (Figure 1A) and the ribD operon that encodes all enzymes for FMN synthesis via termination of transcription (Figure 1B). Riboswitches can sense and bind different types of metabolites, including adenine, guanine and 2′-deoxyguanosine, Mg^2+^, Mn^2+,^ and F^−^, flavine mononucleotide (FMN), S-adenosyl methionine (SAM), TPP, cyclic-AMP, cyclic-di-GMP, glycine, glutamine, lysine, glucosamine-6-phosphate (glmS) riboswitch, etc. [12]. As a result of specific recognition and subsequent ligand binding to the aptamer domain, conformational changes occur along the expression platform via four different mechanisms [9]. Three of these are cis-acting regulatory mechanisms, such as prevention of translation (Figure 1A), transcription termination (Figure 1B), and destabilization of mRNA (Figure 1C), and the fourth mechanism is trans-acting [15,28,29,30,31,32,33,34,35,36,37,38].

Riboswitches regulate the expression of about 7% of bacterial genes [39]. Most of these genes are responsible for synthesizing essential metabolites, without which the cell cannot function. The aptamer domain of the FMN riboswitch is responsible for precisely sensing the concentration of FMN in bacteria [13]. Conformational changes occur when the FMN is bound to the aptamer, resulting in a down-regulation of the expression of all five genes responsible for the synthesis of FMN in bacteria via termination of transcription (Figure 1B). The FMN aptamer of the fmnP gene controls the gene expression of the FmnP protein via the prevention of translation. The FmnP is responsible for FMN import. Therefore, the FMN riboswitch regulates gene expression via transcription termination in the ribD operon or by preventing translation. The FMN aptamer structure resembles a butterfly, with six stems [40,41]. It is located asymmetrically in the binding site, interacting with the chromophore and Mg^2+^-mediated contacts with the phosphate moiety.

The aptamer part of the TPP riboswitch is responsible for the sensation and binding of TPP. It is also known as THI-element or THI-box [42]. It has five stems, and its expression platform controls gene expression via transcription termination or prevention of translation. The formation of a terminator hairpin causes the termination of transcription after TPP binding to the aptamer. The thiamine transport and biosynthesis genes are found in the three operons provided by the riboswitch—thiCEFSGH, thiMD, and thiBPQ [43,44].

The structure of SAM-I riboswitch is formed from four branches connected (via a loop at the end of stem P2 and J3/4 junctions) to two sets of coaxial helixes arranged next to each other [45]. It forms a binding pocket, which is sensitive to SAM levels, from the aptamer part near helixes P1, P2, and J1/2 [18]. The sulfur in the structure recognizes the methyl group, which forms an electrostatic interaction with negative surface potential. Adenine-uracil pairs are highly conservative, and their changes and identity (guanine-cytosine pairs instead of adenine-uracil) reduce the riboswitch’s binding affinity to SAM. In general, riboswitches in Gram-positive bacteria regulate gene expression via transcription termination, while in Gram-positive bacteria, riboswitches work via prevention of translation.

The glmS riboswitch controls gene expression via mRNA destabilization (Figure 1C). This riboswitch works as a ribozyme via self-cleavage induced by an mM concentration of glmS, the highest riboswitch-activating concentration. This riboswitch does not have an aptamer domain. The glmS is a cofactor that binds to the catalytic center of the ribozyme, promoting its self-cleavage.

### 2.2. Distribution of Four of the Most Widespread Riboswitches in Bacterial Pathogens

Distribution is an important consideration for choosing a suitable riboswitch as a target for discovering novel antibacterial drug candidates [39]. Most known riboswitches occur in bacteria and archaea, except TPP riboswitches, which have also been discovered in eukaryotes, including plants and fungi [12]. One riboswitch class can be found in many different organisms, such as bacteria, in repeated multiple copies in a particular genome (Table 1). Riboswitches are widespread in Gram-positive and Gram-negative human-pathogenic bacteria’s genomes and are not found in the human genome [12,13]. The most common riboswitches spread in human-infecting bacteria are TPP, cobalamine, FMN, glycine, SAM-I, lysine, cyclic-di-AMP, ZTP, purines, and glmS riboswitches.

SAM-I, glmS, TPP, and FMN riboswitches are found in the genomes of 60 human-pathogenic bacteria (Table 1). The TPP class of riboswitches is found in the genome of 5624 bacteria, of which 59 infect humans [13,16]. The cobalamine (B12) riboswitch class is found in more than 4914 bacterial types, of which 36 are human-infecting bacterial pathogens [12]. FMN riboswitches are found in 2403 bacterial types, of which 49 are human pathogens (Table 1) [12]. The SAM-I riboswitch and the glmS riboswitch classes are found respectively in 2598 and 912 bacterial species, from which 28 and 31 human-infecting bacterial pathogens (Table 1).

One or more riboswitches are found in all 12 bacteria that 2017 the World Health Organization (WHO) has presented as priority targets for developing new antibacterial agents (Table 1). These bacteria are separated into three categories: critical priority, high priority, and medium priority pathogens for research and development of new antibiotics. *Acinetobacter baumannii*, *Enterobacteriaceae*, and *Pseudomonas aeruginosa* are carbapenem-resistant in the first group [4,46,47]. For example, FMN, glmS, cobalamin, lysine, and other riboswitches are found in *Acinetobacter baumanii*’s genome. This is an excellent prerequisite for each of the presented riboswitches to be subjected to bioinformatics and genomic analyses to determine their suitability for drug targets.

In the second group are *Enterococcus faecium* (vancomycin-resistant), *Staphylococcus aureus* (*S. aureus*) (methicillin-resistant, vancomycin-intermediate and resistant), *Helicobacter pylori* (clarithromycin-resistant), *Campylobacter* spp. (fluoroquinolone-resistant), *Salmonellae* (fluoroquinolone-resistant), and *Neisseria gonorrhoeae* (cephalosporin-resistant and fluoroquinolone-resistant) [48,49,50,51]. For example, in *S. aureus*’s genome, there are even more riboswitch classes, such as TPP, FMN, B12, lysine, glmS, purines, SAM-I, etc. In the third group are *Streptococcus pneumoniae* (penicillin-non-susceptible), *Haemophilus influenzae* (ampicillin-resistant), and *Shigella* spp. (fluoroquinolone-resistant). In the genome of the *Haemophilus influenzae* TPP, FMN, lysine, glmS, etc. riboswitches are found.

Instances of the four riboswitches are found in 60 human-pathogenic bacteria (Table 1), while all 55 different riboswitch classes are found in 68 bacterial pathogens. Thus, with our selection of 4 riboswitches, which make up 7% of all riboswitch classes, we cover 88% of bacterial pathogens where any riboswitch is present. Our latest research has proven that we can use the aptamer part of the four riboswitches from one or more bacterial strains as drug targets for ASOs [15]. The binding of the ASOs to the riboswitch aptamers causes RNase H-mediated degradation of the targeted mRNA(s), which blocks synthesis and the import of an essential bacterial metabolite [14,18,19]. Riboswitches regulate the synthesis of specific and essential metabolites for the cells [16]. If these metabolites are not synthesized, the bacteria cannot survive or will stop their cell division.

## 3. In Silico Analyses of Riboswitches as Antibacterial Drug Targets

Since the discovery of the first riboswitches in 2002, their structures have been well studied. Therefore, it is possible to quickly find each of them in any sequenced bacterial genome with the help of bioinformatics search in databases. It is also easy to find the regulation of the metabolic pathways it is involved in.

Using large databases such as NCBI’s GenBank (https://www.ncbi.nlm.nih.gov/genbank/, accessed on 1 June 2023) and Rfam (https://rfam.org/, accessed on 1 June 2023), it is also possible to assemble libraries and fasta text files with the exact nucleotide sequences of the riboswitches found in the genomes of specific bacterial species [52,53,54]. As a result of multiple alignments and other cluster analyses of the nucleotide sequences, common motifs of the same aptamers found in different bacteria can be selected. They can be targets for potential candidate antibacterial agents.

The diversity of riboswitches in different organisms suggests that some are more suitable for drug targeting than others. Some riboswitches are ideal targets for antibacterial drug discovery, while others are unsuitable. Therefore, to save time and resources by minimizing failed experiments and clinical trials, we have developed a rational system of criteria for assessing riboswitches as antibacterial drug targets, based on which we group them into four separate categories, such as ‘most suitable’, ‘very suitable’, ‘suitable’, and ‘not suitable’ for potential use as targets (Table 2) [13,16].

The riboswitches from the ‘most suitable’ group control the bacteria’s unique and essential biosynthetic pathways and a transporter protein for the key metabolites from the outer environment. The riboswitches from the ‘very suitable’ group control an essential and unique biosynthetic pathway, not the protein transporter for the key metabolites from the outer environment. The riboswitches in the third category control the main biosynthetic pathways, but alternative biosynthetic pathways exist for the same specific metabolite. They are named ‘suitable riboswitches.’ In the fourth category are the ‘unsuitable riboswitches’, which do not control biosynthetic pathways but rather the degradation of metabolites.

We checked distribution via bioinformatics databases to assign each riboswitch of interest to one of the four groups. We grouped those found in the bacterial genomes of human pathogens (Table 2). We applied cluster analyses and multiple alignments to select conservative fragments of their aptamer. This allowed us to design specific ASOs for more than one bacterium or many bacteria. Thus, if the ASO targets a region of mRNA found in many bacteria, it will be a broad-spectrum agent. In contrast, if the targeted region is found in one or few bacteria, it will be a narrow-spectrum antibacterial agent.

The subsequent steps in determining the suitability of riboswitches as drug targets were related to their involvement in various biochemical processes. Do they control the main biosynthetic pathway of an essential metabolite? Is there a specific transporter protein for the metabolite, and is it under the riboswitch’s control? Is there an alternative biosynthetic pathway for the metabolite that is not controlled by the riboswitch [55,56,57]? The ideal, most suitable riboswitches for drug targets have to control the unique biosynthetic pathways of an essential metabolite, without which bacteria cannot function. There must be no alternative biosynthetic pathways for synthesizing the essential metabolite which are not controlled by the riboswitch. In addition, if there is a transporter protein for the metabolite, its expression must also be under the riboswitch’s control.

Such an ideal riboswitch is that for FMN (Table 2). It controls the biosynthetic pathway for FMN and the specific transport of flavin [34,40,41,58]. If the bacterial synthesis of FMN is blocked, oxidation of fatty acids will be ineffective, and the bacterium will be unable to metabolize porphyrin, pentozuron, and glucuronium degradation (Table 2). As the flavin riboswitch controls the expression of all five enzymes necessary for biochemical reactions involved in flavin synthesis, it can be used as a target to block the synthesis of FMN. The FMN riboswitch regulates gene expression via two mechanisms: the termination of transcription (Figure 1B) and the prevention of translation (Figure 1A). The ribDEAHT (ribD) operon, with ypuE, ribD (ribG), ribE (ribB), ribA, ribH, and ribT genes, encodes pyrimidine deaminase, pyrimidine reductase, riboflavin synthase alpha subunit, GTF cyclohydrolase/3,4-dihydroxy-2-butanone-4-phosphate synthase, and the beta subunit of riboflavin synthase enzymes required for riboflavin synthesis (Figure 1B) [13,19]. The second type of genetic control the FMN riboswitch exerts is regulating gene expression via translational initiation in the 45 uraA (ribU), or fmnP, gene, which encodes a putative riboflavin transporter protein (Figure 1A). The SAM-I riboswitch is found upstream of genes that encode enzymes involved in methionine and cysteine biosynthesis in Gram-positive bacteria [12]. The main synthetic pathways for methionine and cysteine production are evolutionarily conservative in many bacteria and are under the genetic control of the SAM classes of riboswitches [18,59,60]. Based on that, FMN and SAM-I riboswitches are part of the most suitable riboswitches as targets for novel drug-designed candidate antibiotics [13]. The glmS riboswitch regulates glmS synthesis and, therefore, peptidoglycan biosynthesis. It has specific control over the gene expression through destabilizing the mRNA via self-cleavage, leading to negative regulation of the synthesis of glmS. There is an alternative pathway for the biosynthesis of glmS, which is not controlled by the riboswitch. Therefore, the glmS riboswitch is in the ‘suitable’ group (Table 2).

The TPP riboswitch is involved in vitamin B1 synthesis. It is a coenzyme involved in carbohydrate metabolism. Thiamine is synthesized by coupling two precursors: 4-amino-5-hydroxymethyl-2-methyl pyrimidine pyrophosphate (NMP-P) and 5-(2-hydroxyethyl)-4-amino thiazole monophosphate (THZ). Another biosynthetic pathway in which the riboswitch is involved is thiamine salvage II. The pyrimidine moiety of thiamine, HMP-PP (under the control of TPP riboswitch), is produced from aminoimidazole ribotide, where the ThiC enzyme produces HMP-P. The thiamine biosynthesis can be inhibited by blocking ThiE synthase by targeting the TPP riboswitch and ThiK kinase with ASO or other candidate antibiotics, causing the death of the bacteria [13].

As a consequence of analyzing the suitability of different bacterial riboswitches as potential targets, we selected the ‘most suitable’, ‘very suitable’, and ‘suitable’ ones in certain bacteria. In some bacteria, the TPP riboswitch is ‘most suitable’, while in others it is ‘very suitable’.

We conducted bioinformatics and genomic analyses with the riboswitches aptamer’s nucleotide sequences. The motifs which we have selected as a result of the multiple alignments and ClustalX analysis were chosen using the Basic Local alignment search tool (BLAST), whether found in the human genome and the genome of other bacteria: pathogenic or probiotic. The BLAST analysis allowed us to select genome fragments of the pathogenic bacterium that do not overlap with those from the human genome or another ‘good’ bacterium from the human microbiome. After the BLAST analysis, inappropriate motifs were excluded, and those that met the requirements were used to create the ASO design. Our selected motifs have a length corresponding to 13–16 nucleotides. Using the program RevComOligo from Prof. Dr. Penchovsky’s official website (https://penchovsky.atwebpages.com/applications.php?page=41, accessed on 1 June 2023), we generated its reverse complementary sequence, which we then checked using the Vienna RNAfold for its binding free energy value.

All these bioinformatics and genomic analyses allowed us to determine the potentially most efficient riboswitches with their relevant aptamer motifs. Thus, with the help of freely available online products, we could perform preliminary analyses that helped us to know how our newly created agents would then affect specific human bacterial pathogens, which metabolites would not be synthesized or imported, and how this would affect the bacterial cell. Thus, we reduced the number of unsuccessful laboratory experiments, time spent on in vitro and in vivo experiments, huge reagent costs, etc.

## 4. Designing Principles of ASO as Antibacterial Agents

Until now, ASOs have been repeatedly used as drug candidates for treating bacterial infections [7,19]. ASOs are unmodified or chemically modified short single-stranded nucleic acids that bind specifically to targeted RNA or DNA. The principles by which ASOs exert their activity are RNase H-dependent, Rnase P-dependent, and six different Rnases-independent, including no-go degradation, blocking miRNA recognition elements, miRNA sequestration, increasing protein expression, sequestered protein release, and splicing modifications [61,62,63,64]. In the Rnase H-dependent mechanism, ASOs remain unchanged, while the Rnase H enzyme cleaves the targeted mRNA, preventing its translation and, therefore, specific protein expression. As a result of enzymatic cleavage via Rnase H, the expression of proteins with an important effect on the survival and division of the bacterium is inhibited [65,66,67]. The ASO function depends on whether it will reach the tissues and cells associated with a certain disease and whether it will pass through the cell membrane and cause an inhibition of the targeted RNA. We have attached the universal CPP, pVEC, to our ASOs to deliver highly efficient ASOs into the cell (Table 3). pVEC is a universal CPP because it can enter eukaryotic and prokaryotic cells, including Gram-positive and Gram-negative bacteria. Therefore, we can tackle both extra- and intracellular bacterial infections.

ASOs are extremely versatile due to their chemical modifications [20,68,69,70,71,72]. Several generations have been created, possessing different characteristics, advantages, and disadvantages. ASOs are increasingly built by combining the first and second generations of chemical modifications to achieve greater efficiency and stability, prolonging the ASO’s half-life and specific targeting [20,55,70,71].

The first generation of ASO modifications is characterized by the activation of the endonuclease enzyme rNase H [69]. It recognizes the formed double-stranded regions between the ASO and the RNA target and cleaves the RNA molecule without affecting the ASO. Thus, the ASO can bind another mRNA and requires micromolar or nanomolar concentrations to achieve an effect, working under multiple-turnover conditions. However, ASOs from the first generation do not have sufficient stability and specificity in the cell. Therefore, there is a high possibility of non-specific binding to the SH groups in the proteins on the cell’s surface or inside. In this way, unwanted cytotoxicity may be induced.

In the second generation of modifications, one hydrogen atom at the 2’-O position of the pentose ring of the nucleotide is replaced with methyl or other groups [73]. That modification increases the ASO’s stability against endo- and exonucleases. It reduces the non-specific binding to proteins compared to the PS-modification, increasing binding affinity to the targeted complementary RNA. If the ASO is designed to have both types of modifications, the plasma half-life and uptake into tissues and cells of the ASO are increased. All of the mentioned ASOs in the article are chimeric ASOs with modifications from the first and the second generations.

The third generation of ASOs includes a more diverse group of chemical modifications. They have chemical modifications that are much more resistant to nucleases than the first generation. The third generation of ASOs includes peptide nucleic acid (PNA) and locked nucleic acid (LNA), which bind to RNA and DNA much more strongly than the same sequences of dsRNA or dsDNA [74,75,76]. The second and third generations of ASOs do not activate RNA-mediated cleavage, unlike the first generation of ASOs.

The design of the different ASOs has implemented essential criteria as follows [19]:

1. The target RNA domain must be strictly single-stranded and accessible for hybridization with the ASO. The formed ASO/RNA hybrid must be stable.
2. There are no significant similarities with the expressed coding of human RNAs.
3. ASO does not form a stable secondary structure and is accessible to complementary hybridize with the target (single-stranded RNA part.
4. ASO does not create stable double-stranded hybrids.
5. To ensure the penetration of ASOs into the bacteria, the CPP pVEC is attached to them [22,23].
6. The PS-modified nucleotides of ASOs are not recommended to be above 10 nt due to the increased risk of non-specific binding to SH-group-containing peptides.
7. ASOs are not recommended to be above 22 nt due to the reduced cellular uptake.

Most ASOs without a carrier cannot pass through the cell barrier of the target cells, especially if they are bacterial [24,27,49]. Therefore, we have chosen the CPP pVEC as a well-established penetrator in prokaryotic and eukaryotic cells. It is an oligopeptide composed of 18 amino acid residues (LLIILRRRIRKQAHAHSK), derived from the murine vascular endothelial-cadherin protein VE-cadherin. The pVEC is connected covalently to the ASO.

In compliance with all requirements for the rational design of ASOs, a 100% success rate for their antibacterial effect is guaranteed. For FMN targeting, we have created pVEC_FMN_ASO_1, which is reverse complementary to the sequence of the aptamer domain from the FMN riboswitch, where the index ‘_1_‘ refers 2′-alkyl modifications of the ribose, and index ‘_2_’ refers to phosphorothioate (PS) linkage (Table 3) [19].

To target the TPP riboswitch in *S. aureus*, pVEC_TPP_ASO_1 has been designed (Table 3) [14]. To target the SAM-I riboswitch found in *S. aureus* and *Listeria monocytogenes* (*L. monocytogenes*), we designed pVEC_SAM-I_ASO_1 (Table 3) [18]. To inhibit glmS synthesis, we have created two ASOs: pVEC_glmS_ASO_1 and pVEC_glmS_ASO_2 (Table 3) [17]. The first one has a length of 16 nucleotides and is designed to target the glmS ribozyme, while the second targets the nagA mRNA. Both are specific to *S. aureus*.

Until now, only some riboswitches have been tested with candidate antimicrobial agents. Thus, it is assumed that bacteria have not developed neutralization or alternative synthesis mechanisms against agents targeting riboswitches. However, upon application of ASOs, the bacterium can develop insensitivity to the ASO by mutating the aptamer sequence. In this case, it will be easy to sequence the mutated aptamer part and redesign the ASO accordingly.

## 5. Targeting Bacterial Riboswitches with ASO for Antibacterial Development

We have tested various ASOs that target glmS, FMN, TPP, and SAM-I riboswitches as antibacterial agents. We have applied the rational criteria for selecting our targets and designing our ASOs as described above.

### 5.1. glmS Riboswitch

**Figure 2:**
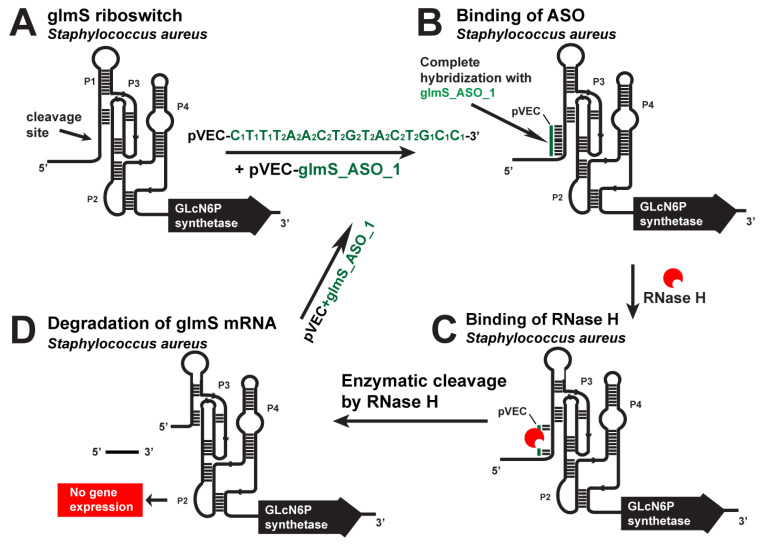
Targeting the *S. aureus* glmS riboswitch with a specific chimeric antisense oligonucleotide. (**A**) The chimeric pVEC_glmS_ASO_1 complex with the cell-penetrating oligopeptide pVEC binds to the complementary sequence of the glmS aptamer domain. (**B**) After their binding, a double-stranded molecule is formed. (**C**) The double-stranded molecule is recognized by RNase H, which binds it and triggers the enzymatic cleavage of the glmS mRNA. (**D**) The enzymatic cleavage causes a degradation of the glmS mRNA part and, as a result, prevents gene expression and glmS synthesis.

The binding of pVEC_glmS_ASO_1 (Table 3) with the complementary sequence of the glmS riboswitch of *S. aureus* leads to enzymatic degradation of the glmS mRNA by the endonuclease RNase H (Figure 2). Bioinformatics research has found two biochemical pathways for synthesizing the essential metabolite glmS. When glucose enters the cell, it is converted into glucose-6-phosphate (Glc-6-P), which is converted into fructose-6-phosphate (Fru-6-P). This process is carried out with fructose-6-phosphate aminotransferase, regulated by the glmS riboswitch. Fru-6-P is part of glycolysis. The enzyme glucosamine-6-phosphate (GlcN6P) deaminase, encoded by the gene nagB, catalyzes the reverse reaction of GlcN6P to Fru-6-P. When the deamination reaction takes place, ammonia is released. The imported glucosamine that has already been taken up inside the cell with ATP consumption is converted into GlcN6P. nagA regulates the deacetylation of N-acetylglucosamine-6-phosphate by N-acetylglucosamine-6-phosphate deacetylase. It is an alternative pathway for the synthesis of glmS.

**Figure 3:**
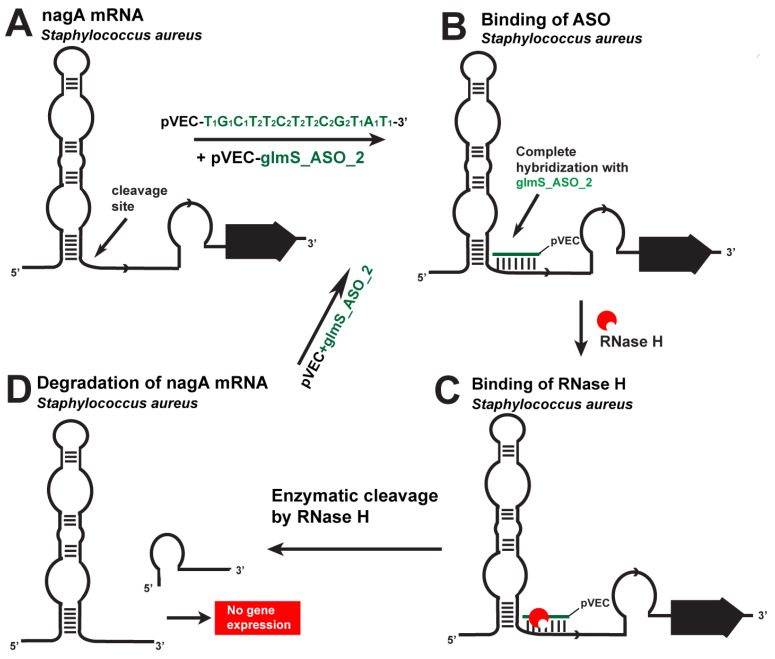
Targeting *S. aureus* nagA mRNA with a specific ASO. (**A**). The chimeric pVEC_glmS_ASO_2 binds to the complementary sequence of nagA mRNA. (**B**). After their binding, a double-stranded molecule is formed. (**C**). The double-stranded molecule is recognized by RNase H. (**D**). The targeted nagA mRNA is cleaved.

pVEC_glmS_ASO_1 targets the glmS riboswitch (Figure 2), while pVEC_glmS_ASO_2 targets the nagA mRNA (Figure 3). When combined, the two ASOs block the synthesis of glmS entirely and kill S. aureus. The reverse conversion of the PlcN-1-P to GlcN6P is possible, but since it has no alternative pathways for the synthesis of the GlcN1P, it remains entirely dependent on the transformation of GlcN6P. Studies have shown that the nagA, nagB, glmS, and glucosamine kinase genes are essential in distributing sugars to cell wall synthesis and glycolysis in bacteria. The most important of them are glmS and nagA. When they are blocked, cell-wall synthesis is inhibited. That leads to a bacteriocidic effect.

The inhibitory effect of pVEC_glmS_ASO_1 has been tested against three different bacteria, including human-pathogenic bacteria *S. aureus* and *Escherichia coli* (*E. coli*) and the nonpathogenic bacterium *Bacillus subtilis* (*B. sublilis*). Its design is specific only to the glmS riboswitch of *S. aureus*. Clustal analysis showed mismatches of the riboswitches sequences in *E. coli* and *B. subtilis*. As a result, the pVEC_glmS_ASO_1 does not specifically bind their mRNAs.

After isolation of total RNA, converted into cDNA via reverse transcriptase and amplified via PCR, the results demonstrated that the pVEC_glmS_ASO_1 successfully inhibits the glmS RNA. Its inhibitory effect was also demonstrated after 0, 150, 350, 700, 1000, and 2000 nM of ASO. The pVEC_glmS_ASO_1 showed maximum inhibition of the *S. aureus* growth at 2000nM concentration. At the same concentration, there was no effect on *E. coli* and *B. subtilis* growth. The two lowest concentrations of pVEC_glmS_ASO_1 do not inhibit the growth of *S. aureus*. The pVEC_glmS_ASO_1′s minimum concentration for 80% inhibition (MIC80) of *S. aureus* is 700 nM or 5 μg/mL.

The laboratory experiments continued with a microbiology test on a Petri dish with LB agar serving as a control, which was inoculated with 50 μL of a 5 h-grown culture of *S. aureus* and cultured overnight at 37 °C. Another Petri dish was inoculated with 50 μL of a 5 h-grown culture of the bacterium, treated with a combination of 1000 nM pVEC_glmS_ASO_1 and 1000 nM pVEC_glmS_ASO_2 overnight at 37 °C. The results on the second Petri showed that pVEC_glmS_ASO_1 and glmS_ASO_2 killed *S. aureus*. The toxicity test demonstrated that the pVEC is not toxic to the growth of *S. aureus* at a concentration of 2000 nM, which automatically proves the effect of the ASO. Without pVEC, the designed ASO cannot pass through the bacterial cell membrane (at concentrations of 150, 350, 700, 1000, and 2000 nM). As a result, there is no inhibitory effect, which has also been demonstrated in vitro.

In conclusion, the results demonstrate that glmS riboswitch is a suitable target, and pVEC_glmS_ASO_1 has a bacteriostatic effect because it stops the main pathway for the synthesis of glmS in *S. aureus*. When both pVEC_glmS_ASO_1 and pVEC_glmS_ASO_2 are applied, we observed a bacteriocidic effect because the synthesis of glmS was completely stopped. These ASOs work as narrow-spectrum antibiotics.

### 5.2. FMN Riboswitch

**Figure 4:**
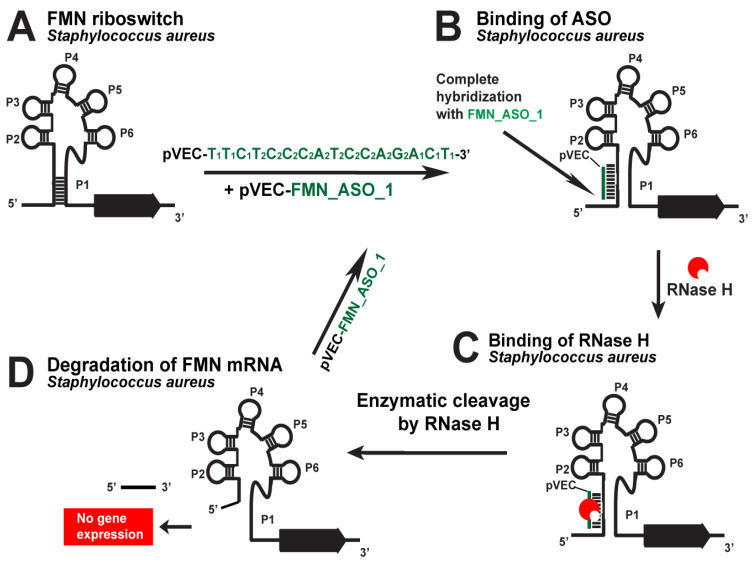
Targeting the *S. aureus* FMN riboswitch with a specific chimeric antisense oligonucleotide. (**A**) The chimeric ASO pVEC_FMN_ASO_1 complex with pVEC binds to the complementary sequence of the FMN aptamer in *S. aureus*. (**B**) A double-stranded molecule is formed after the ASO and mRNA binding. (**C**) RNase H recognizes the double-stranded molecule and triggers the enzymatic cleavage of mRNA. (**D**) The enzymatic cleavage of mRNA leads to no gene expression of the ribD operon, and this causes inhibition of bacterial growth.

The FMN riboswitch is one of the most widespread riboswitches in bacteria (Table 1). It has a highly conserved RNA in the 5′-UTR of prokaryotic mRNAs coding many enzymes for FMN synthesis and a protein responsible for flavin import. pVEC_FMN_ASO_1 targets the ribD operon and ypaA gene in *S. aureus*, *E. coli*, and *L. monocytogenes* (Figure 4).

pVEC_FMN_ASO_2 has eight mismatches in its sequence compared to pVEC_FMN_ASO_1 and serves as a negative control, demonstrating the effectiveness of pVEC_FMN_ASO_1. When the complex of pVEC_FMN_ASO_1 enters one of the bacteria, the oligonucleotide part of the ASO specifically hybridizes to the aptamer domain of the FMN riboswitch. Complementarily bound, they form a double-stranded molecule, which is recognized and bound by the RNase H endonuclease, and leads to enzymatic hydrolysis of ribD and ypaA mRNAs, thus blocking the gene expression of the ribD operon and ypaA gene. The experiments showed that in the samples with the 2000 nM concentration of pVEC_FMN_ASO_1, there is maximum inhibition of *S. aureus*, *E. coli*, and *L. monocytogenes* growth.

*L. monocytogenes* growth with pVEC_FMN_ASO_1 reaches a maximum of around 0.3 OD after 4 h incubation and stays the same over the next 8 h. The *E. coli* growth in the presence of pVEC_FMN_ASO_1 reaches a maximum of around 0.3 OD after 4 h incubation and stays the same over the next 8 h. The *S. aureus* growth reached a maximum of around 0.2 OD after an incubation time of three and a half hours and stayed the same over the next 8 h. In the presence of pVEC_FMN_ASO_2, which does not bind specifically to the domain of *E. coli* and *L. monocytogenes*, they reached a maximum of 1.3 OD after incubation for 4 h. They stayed the same over the next 8 h. With pVEC_FMN_ASO_1, *E. coli* and *L. monocytogenes* growth reaches a maximum of less than 0.4 OD after 4 h of incubation without change for the next 8 h. In the presence of pVEC_FMN_ASO_1, *S. aureus* growth reaches a maximum of less than 0.3 OD after an incubation of three and a half hours. When the ASO concentration is 700 nM, the growth inhibition of the three bacteria is the same as in the previous two higher concentrations. In the presence of pVEC_FMN_ASO_1, *E. coli* and *L. monocytogenes* reach a maximum of 1.3 OD after four and a half hours of incubation, and *S. aureus* 1.1 OD after 4 h of incubation. The MIC80 for pVEC_FMN_ASO_1 is around 700 nM, 4.5 μg/mL for the three bacteria, causing a bacteriostatic effect. Subsequent analyses confirmed that the observed inhibitory effects were due solely to the effectiveness of the ASO, and the cell-penetrating peptide did not exhibit bacterial toxicity (in concentrations ranging from 0 to 2000 nM). Without pVEC, FMN_ASO_1 cannot enter the bacterial cell and is ineffective.

The toxicity test showed that pVEC_FMN_ASO_1 is not toxic to the human cell lining of non-small cell lung cancer A549 at a concentration of 750 nM, 4.5 μg/mL. When pVEC_FMN_ASO_1 reaches a concentration of 2000 nM, the survival of the A549 human cell line is 68%, with only 32% toxicity. When pVEC_FMN_ASO_1 concentration is 1125 nM, the survival of the A549 human cell line is 98%, with only 2% toxicity. In conclusion, the results demonstrate that the FMN riboswitch is a suitable target in antisense technology for antibacterial drug development, as pVEC_FMN_ASO_1 is a suitable candidate for antibacterial agents. pVEC_FMN_ASO_1 works as a broad-spectrum antibacterial agent that targets and stops both the synthesis and the transport of FMN in bacteria.

### 5.3. TPP Riboswitch

**Figure 5:**
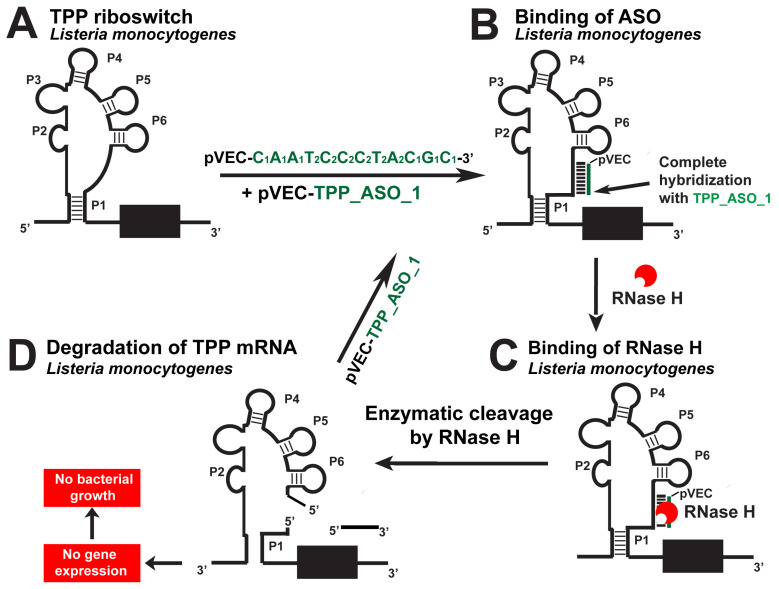
Targeting the *L. monocytogenes* TPP riboswitch with a specific chimeric antisense oligonucleotide. (**A**) The chimeric antisense oligonucleotide pVEC_TPP_ASO_1 binds to the complementary sequence of the TPP aptamer of *L. monocytogenes*’ riboswitch. (**B**) A double-stranded molecule is formed after the ASO and mRNA binding. (**C**) RNase H recognizes the double-stranded molecule and triggers the enzymatic cleavage of mRNA. (**D**) The enzymatic cleavage of mRNA leads to no gene expression of the three thi-operons, and this causes inhibition of bacterial growth.

The TPP riboswitch is the most widespread in the genomes of all bacteria, particularly in human-pathogenic bacteria (Table 1) [12]. According to the Rfam database, it is found in the genome of 59 human-pathogenic bacteria (Table 2). The chimeric pVEC_TPP_ASO_1 (Table 3) is designed to target the aptamer domain of TPP riboswitch found in *L. monocytogenes* and *B. subtilis* (Figure 5) [14]. When bound to the specific targets, it enables the mRNA to be cleaved by RNase H under multiple turnover conditions. RNase H hydrolyzes the targeted RNA, leading to a lack of mRNA translation. As a result, it blocks the gene expression of enzymes, which are part of the thiamine biosynthesis.

Different concentrations of pVEC_TPP_ASO_1 were tested during the laboratory tests, including 0, 150, 350, 700, 1000, and 2000 nM, of pVEC_TPP_ASO_1. The maximum inhibitory effect was established in the samples containing the highest concentration of pVEC_TPP_ASO_1. Observations of the growth of an *L. monocyte* gene treated with 2000 nM pVEC_TPP_ASO_1 reached a maximum of less than 0.3 OD after 4 h. The maximum effect reached over the *B. subtilis* growth with 2000 nM pVEC_TPP_ASO_1 was reached at 0.3 OD after 4 h incubation and did not change during the following 8 h. In the presence of pVEC_TPP_ASO_1, the growth of *L. monocytogenes* and *B. subtilis* reached a minimum of 0.1 OD after 4 h. The bacterial growth of *L. monocytogenes* reached a maximum of 0.2 OD after 4 h of incubation and stayed the same over the following hours with 1000 nM pVEC_TPP_ASO_1. The bacterial growth of *B. subtilis* reached a maximum of around 0.4 OD after 4 h of incubation. The controls, without pVEC_TPP_ASO_1, showed that the bacterial growth of *L. monocytogenes* and *B. subtilis* reached a maximum of 1.3 OD after 4 h.

With 700 nM TPP_ASO_1, the bacterial growth of *L. monocytogenes* and *B. subtilis* reached a maximum of 0.4 OD after 4 h of incubation. Inhibition of the bacterial growth of *L. monocytogenes* and *B. subtilis* was not observed in the samples with a lower concentration of pVEC_TPP_ASO_1 (350 nM and lower).

The MIC80 for pVEC_TPP_ASO_1 is 700 nM, 5 μg/mL. pVEC is not toxic for all tested cells in all concentrations from 0 to 2000 nM. The toxicity test proved that 700 nM pVEC_TPP_ASO_1 is not toxic to the A549 human non-small lung cancer cell line. When the concentration of ASO is 1000 nM, the survival of the A549 human cell line is 98.5%. In the presence of 1000 nM pVEC_TPP_ASO_1, the survival of the A549 human cell line was 82%.

The effect of pVEC_TPP_ASO_1 was also tested on *Escherichia coli*. Results showed that it does not affect bacterial growth because it is not specifically designed to target *E. coli*, since the related riboswitch is not present there. After all the values shown, we can conclude that the specifically created pVEC_TPP_ASO_1 is a promising antibacterial candidate agent with a bacteriostatic effect on *L. monocytogenes*.

### 5.4. SAM-I Riboswitch

**Figure 6:**
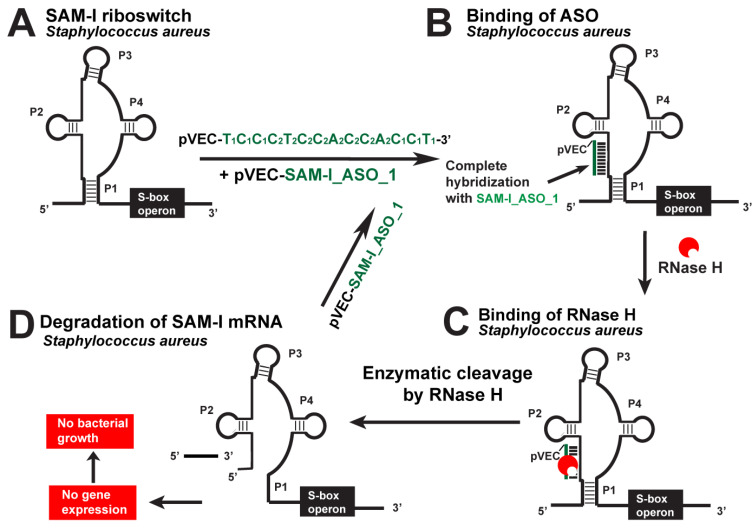
Targeting the *S. aureus SAM-I* riboswitch with a specific chimeric antisense oligonucleotide. (**A**) The chimeric pVEC_SAM-I_ASO_1 binds to the complementary sequence of the SAM-I aptamer domain of *S. aureus’* riboswitch. (**B**) A double-stranded molecule is formed after the ASO and mRNA binding. (**C**) RNase H recognizes the double-stranded molecule and triggers the enzymatic cleavage of mRNA. (**D**) The enzymatic cleavage of mRNA leads to no gene expression of the S-box operon, and this causes inhibition of bacterial growth.

The SAM-I riboswitch was targeted with the pVEC_SAM-I_ASO_1 in *S. aureus* and *L. monocytogenes* as one of the most promising riboswitch targets (Figure 6). In the absence of pVEC_SAM-I_ASO_1, *L. monocytogenes* reached a maximum of about 1.3 OD. In the presence of 2000 nM pVEC_SAM-I_ASO_1, *L. monocytogenes* reached a maximum of less than 0.3 OD after incubation of 4 h [18].

In the absence of pVEC_SAM-I_ASO_1, the growth of *S. aureus* reached a maximum of 1.3 OD after 4 h of incubation. In the presence of 2000 nM pVEC_SAM-I_ASO_1, the growth of *S. aureus* reached a maximum of 0.3 OD after 4 h incubation [18]. At the concentration of pVEC_SAM-I_ASO-1 of 700 nM, inhibition of the bacterial growth of *L. monocytogenes* and *S. aureus* was observed to be the same as in the previous two concentrations. With 700 nM pVEC_SAM-I_ASO_1, the growth of *L. monocytogenes* reached 0.5 OD after 4 h of incubation.

With 700 nM pVEC_SAM-I_ASO_1, the growth of *S. aureus* reached a maximum bacterial growth of around 0.4 OD after 3.5 h of incubation.

At the 150 nM pVEC_SAM-I_ASO_1 concentration, the *S. aureus* and *L. monocytogenes* bacterial growth was not inhibited. With the ASO, both bacteria reached the maximum of 1.3 OD after 4 h of incubation.

The SAM-I riboswitch is not found in the genome of *E. coli.* Because of that, experiments with the ASO and that bacteria have been conducted as a negative control test. The highest concentration of pVEC_SAM-I_ASO_1, 2000 nM, was incubated with *E. coli*, and the bacterial growth was not inhibited.

The MIC80 of pVEC_SAM-I_ASO_1 for *S. aureus* and *L. monocytogenes’s* growth inhibition is 700 nM (4.5 µg/mL). At that concentration, pVEC_SAM-I_ASO_1 does not cause toxicity in the human cell line A549, which is derived from non-small cell lung cancer. At a concentration of 2000 nM pVEC_SAM-I_ASO_1, the survival of the A549 human cell line is 61%, with only 39% toxicity. At a concentration of 1000 nM pVEC_SAM-I_ASO_1, the survival of the A549 human cell line is 94%, with only 6% toxicity.

## 6. Materials and Methods

### 6.1. Bioinformatics and Genomics Analysis

Various bioinformatics and genomics analyses have been used to precisely assess the suitability of 55 riboswitch classes as antibacterial drug targets [13,16]. We selected the TPP, FMN, glmS, and SAM-I riboswitches to serve as targets for chimeric ASOs newly designed by our team.

The metabolic pathways in which FMN, TPP, GlmS, and SAM-I are involved have been established in the KEGG PATHWAY (Kyoto Encyclopedia of Genes and Genomes: https://www.genome.jp/kegg/pathway.html, accessed on 1 June 2023) and BioCyc databases (https://biocyc.org/, accessed on 1 June 2023). The nucleotide sequences of the FMN, TPP, GlmS, and SAM-I riboswitches present in various human-pathogenic bacteria have been taken from the Rfam database 13.0 (http://rfam.xfam.org/, accessed on 1 June 2023) and RSwitch database, available online (https://penchovsky.atwebpages.com/applications.php?page=58, accessed on 1 June 2023). Nucleotide sequences have been recorded in fasta files, which were further subjected to multiple sequence and profile alignments with ClustalX (2.0) (http://www.clustal.org/clustal2/, accessed on 1 June 2023) to select specific conserved sequences. The selected conservative motifs have been subjected to further BLAST analyses (Basic Local Alignment Tool: https://blast.ncbi.nlm.nih.gov/Blast.cgi, accessed on 1 June 2023). The main goal of these analyses was to check whether the selected motifs are found in other bacteria or the human genome. If it had been found in human RNA, the motif was rejected. When selected, the motif was a prime target for generating a specific complementary ASO. The Vienna RNA secondary structure server at http://rna.tbi.univie.ac.at (accessed on 1 June 2023) and the RNA alifold program were used to predict their secondary structures. During the design of the ASOs against the four different bacterial targets, the reverse complementary motif was connected to a pVEC attached to the chimeric oligomers 5′-terminus at its carboxyl terminus (Table 3).

### 6.2. Bacterial Strains and Culture Conditions

The bacterial strains used during the laboratory tests were *S. aureus* strain ATCC 25923, *E. coli* strain K1, *B. subtilis* strain 168, and *L. monocytogenes* strain ATCC 8932, purchased from the DSMZ German Collection of Microorganisms and Cell Cultures GmbH, Braunschweig, Germany (https://www.dsmz.de/, accessed on 1 June 2023). The bacteria were cultivated in a Luria−Bertani (LB) medium containing 10 g Bacto tryptone, 5 g yeast extract, and 10 g NaCl per 1 L at pH 7.5. The bacteria were incubated overnight at 37 °C until reaching an optical density of 0.8 units at 600 nm. They were diluted 200 times and incubated at 37 °C with shaking for 12 h with or without an ASO at various concentrations, and the optical density at 600 nm was measured every 30 min. Six different concentrations for each ASO were used, including 2000, 1000, 700, 350, and 150 nM and without any ASO. Three repetitions were performed for each concentration, and the average values were used.

### 6.3. Toxicity of ASO

The toxicity of ASOs was tested in a human cell line derived from non-small cell lung cancer, A549. The cell line was cultured as a monolayer culture (D-MEM medium) with added penicillin (100 U/mL), streptomycin (100 μg/mL), and 10% fetal calf serum. The cells from the A549 line were seeded in a 96-well plate (at 100,000 per well), and the ASOs were added to the culture medium 24 h after their culturing in the exponential growth phase. The cell survival was recorded at the 48th hour of the cell treatment with ASO via the MTT test, according to Mosmann.

## 7. Discussion

AR is a global problem that occurs naturally because of many bacterial mechanisms that have evolved survival strategies against all known antibiotics and because of misuse of antibiotics. The widespread MDR pathogenic bacteria exponentially increase the morbidity and mortality of patients of every age and in every part of the world. This imposes the immediate need to discover new targets in the bacterial genome and new approaches to develop novel antibiotics at a much higher rate.

Various RNAs have proven suitable targets for novel drug discovery. One of the most promising RNA targets is bacterial riboswitches. They are widespread in bacteria but are not found in the human genome. Another advantage is that bacterial pathogens have not encountered therapeutics targeting riboswitches apart from roseoflavin and, therefore, have not developed resistance. Riboswitches are well studied and can be used to select a conserved region with an important regulatory function for synthesizing essential metabolites. We can block the synthesis of such essential metabolites by targeting specific mRNA with ASOs. If the bacterium cannot obtain these metabolites by importing them from the outside or via another alternative metabolic pathway, it will be fatal. In that case, bacteria will suffer and either stop dividing or self-destruct. Riboswitches as targets have been evaluated and proven effective against a list of 19 antimicrobial compounds targeting a specific class of riboswitches and exhibiting antimicrobial activity for each indicated organism (Table 4).

Roseoflavin, the natural analog of riboflavin produced by *Streptomyces davawensis*, binds to the FMN riboswitch with similar to the FMN affinity and prevents the expression of downstream genes in *B. suntilis*, *E. faecalis*, *L. monocytogenes*, and *S. pyogenes*. Another six compounds have been targeted to the same riboswitch. The most promising antimicrobial agent is pVEC_FMN_ASO_1.

If alternative biosynthetic pathways or transport of the key metabolite for the bacterium exist, it can easily be verified whether the same riboswitch controls them. If not controlled by a riboswitch, simple microbiological laboratory tests can assess whether the amount of alternative synthesized or imported metabolite will be sufficient for bacterial survival. This is the case with the glmS riboswitch, where the antimicrobial agent pVEC_glmS_ASO_1 has been tested. Still, for the complete inhibition of bacterial growth and the death of the bacterium, it is also possible to apply a blockade of the alternative pathway to synthesize the key metabolite under the control of the *nagA* gene. For that, we apply a second ASO, pVEC_glmS_ASO_2, for complete inhibition of glmS synthesis (Table 4).

The small molecule PKZ18 has shown an inhibitory effect against Gram-positive and Gram-negative bacteria. In vitro and in vivo experiments have proved that PKZ18 binds to the specifier loop of both tyrS and glyQS TPP riboswitch, acting against multiple TPP riboswitches, and after 24-h exposure, do not show any toxicity in eukaryotic cell lines [91]. PKZ18-22 significantly affects the expression of 8/12 TPP-regulated genes on *S. aureus* multi-resistant strains. Some results showed resistance [39] not seen when targeting the TPP riboswitch with pVEC_TPP_ASO_1 [14].

At present, three different generations of ASOs have been developed. Each has specific modifications that protect them from nucleases and degradation. The PS modifications are typical for the first generation. They induce cleavage of the targeted RNA via RNase H. The 2′-alkyl modification of the ribose, typical for the second generation of modifications, increases the ASO’s resistance to the nucleases in the cell, improves binding affinity, increases efficacy, and decreases the non-specific protein binding of oligonucleotides but inhibits the function of RNase H. The third generation of modifications provides more stability and resistance against enzymatic degradation but inhibits RNase H activity and has a single turnover.

For these reasons, our team chose to work with ASOs with modifications from the first two generations, as the PS modifications are in the middle, flanked by 2′-CH_3_-O- modifications at both terminuses. The designer ASOs in this article are chimeric ASOs, which possess a higher stability and induce the activation of the RNase H, resulting in the cleavage of the targeted RNA under multiple turnovers (Table 4). In addition, to ensure the successful and efficient penetration of the substance into the cell, the CPP pVEP was attached to the 5′-terminus of all ASOs. It provides delivery of the ASO agent without itself inducing toxicity in the bacterium or the human. Due to its structure and origin, it is easily removed from the body.

The nucleotide part of the ASO binds to their specific complementary parts of the riboswitches’ aptamers. RNase H cleaves the double-stranded structure, where the PS-modifications a genetic control on the genes, which are part of the riboswitches’ expression platforms. This prevents translation of the targeted mRNA, and the essential metabolites for the bacteria are not synthesized.

The targeted aptamers of the glmS, FMN, TPP, and SAM-I riboswitches are well studied. We target the conservative part of the riboswitch aptamers that will not mutate easily without disturbing its function. Thus, bacteria cannot easily develop resistance. If, however, the bacterium develops resistance, it will be easy to capture it since there will be mutations in the targeted part of the mRNA. We will easily modify the design of the ASO to the mutated RNA. It is unbelievable that the bacterium can invent enzymes capable of hybridizing the modified ASO or becoming impenetrable for the pVEC.

BLAST analyses ensure that the target region we select is not part of the human genome. Thus, we avoid the possibility of unwanted complementary binding of our ASO to human RNAs. Even now, as in vitro tests for toxicity, we test each of the designed ASOs on the A549 human cell line of non-small cell lung cancer. The results proved that at the MIC80′s dosage, none of the tested ASOs caused any toxicity [14,17,18,19]. All of the tested ASOs showed MIC80 at the same concentration.

We think future improvements to our approach can be achieved using CPPs that enter only bacterial cells but not humans. This can reduce the toxicity of ASOs and give us more flexibility in choosing the target sequence. Probing some different types of ASO modifications may improve the efficiency of inhibition. Another research avenue is investigating the emergency rate of AR against our ASOs.

## 8. Conclusions

Based on postulated and carefully selected criteria, glmS, FMN, TPP, and SAM-I riboswitches were classified as suitable targets for drug discovery. The ASOs with modifications from the first and second generations were specifically designed to target the four riboswitches and to avoid hybridization with human RNAs.

All designed ASOs worked as expected, proving the high fidelity of our rational approach to drug design, including the estimation of drug targets. We can conclude that our bioinformatics methodology for selecting suitable targets works precisely. It allows us to select suitable targets that can then be used in laboratory assays. It also allows us to easily create a specific design of ASO, which will be aimed at a specific target. The approach demonstrated 100% accuracy in four different procedures until now in both parts, RNA target selection and ASO design [14,17,18,19]. It saves a lot of time. The approach is universal, applicable to any RNA target for antibacterial drug development, and easily adaptable to AR. Due to bioinformatics and genomic analyses applied, we can develop ASOs to target one or more bacteria. In this way, we could create either specific narrow-spectrum candidate antibacterial agents or broad-spectrum therapeutics.

## Figures and Tables

**Table 1:** Distribution of four of the most widespread riboswitches in human-infecting bacterial pathogens used as targets of ASOs for antibacterial drug development. The bacteria highlighted are part of the Global Priority Pathogens List of the World Health Organization for the timely and immediate development of new antibacterial candidate drugs. They are classified into three categories: critical priority (+++), high priority (++), and medium priority pathogens (+).

Human Pathogenic Bacteria		glmS Riboswitch	FMN Riboswitch	TPP Riboswitch	SAM-I Riboswitch
1	*Acinetobacter baumannii* +++	+	+	-	-
2	*Actinomyces israelii*	-	+	+	+
3	*Bacillus anthracis*	+	+	+	+
4	*Bacillus cereus*	+	+	+	+
5	*Bacteroides fragilis*	-	-	+	+
6	*Bartonella henselae*	-	-	+	+
7	*Bartonella quintana*	-	-	+	+
8	*Bordetella pertussis*	+	+	+	+
9	*Brucella abortus*	+	+	+	+
10	*Brucella canis*	+	+	+	+
11	*Brucella melitensis*	+	+	+	+
12	*Brucella suis*	+	+	+	+
13	*Campylobacter jejuni* ++	-	-	+	-
14	*Chlamydia pneumoniae*	-	-	+	-
15	*Chlamydia psittaci*	-	-	+	-
16	*Chlamydia trachomatis*	-	-	+	-
17	*Clostridium botulinum*	+	+	+	+
18	*Clostridium difficile*	+	+	+	+
19	*Clostridium perfringens*	+	+	+	+
20	*Clostridium tetani*	+	+	+	+
21	*Corynebacterium diphtheriae*	+	+	+	-
22	*Enterococcus faecalis*	+	+	+	-
23	*Enterococcus faecium* ++	+	+	+	-
24	*Enterobacter* sp. *+++*	+	+	+	-
25	*Escherichia coli* +	+	+	+	-
26	*Francisella tularensis*	+	+	+	-
27	*Haemophilus influenzae* +	+	+	+	-
28	*Helicobacter pylori* ++	-	-	+	-
29	*Klebsiella pneumoniae* +	+	+	+	+
30	*Legionella pneumophila*	-	+	+	-
31	*Leptospira interrogans*	-	+	+	-
32	*Leptospira noguchii*	-	+	+	-
33	*Leptospira santarosai*	-	+	+	-
34	*Leptospira weilii*	-	+	+	-
35	*Listeria monocytogenes*	+	+	+	+
36	*Mycobacterium leprae*	-	+	+	-
37	*Mycobacterium tuberculosis*	-	+	+	+
38	*Mycobacterium ulcerans*	-	+	+	+
39	*Mycoplasma pneumoniae*	-	+	+	-
40	*Neisseria gonorrhoeae* ++	-	-	+	+
41	*Neisseria meningitidis*	-	-	+	+
42	*Nocardia asteroides*	-	+	+	+
43	*Pseudomonas aeruginosa* +++	-	+	+	+
44	*Rickettsia rickettsii*	-	+	+	+
45	*Salmonella enterica* ++	+	+	+	-
46	*Salmonella typhi* ++	+	+	+	-
47	*Shigella dysenteriae* +	-	+	+	-
48	*Shigella sonnei* +	-	+	+	-
49	*Staphylococcus aureus* ++	+	+	+	+
50	*Staphylococcus epidermidis*	+	+	+	+
51	*Staphylococcus saprophyticus*	+	+	+	-
52	*Streptococcus agalactiae*	-	+	+	-
53	*Streptococcus mutans*	-	+	+	-
54	*Streptococcus pneumoniae* +	-	+	+	-
55	*Streptococcus viridans*	+	+	+	+
56	*Streptococcus pyogenes*	-	+	+	-
57	*Vibrio cholerae*	+	+	+	-
58	*Yersinia enterocolitica*	+	+	+	-
59	*Yersinia pestis* +	+	+	+	-
60	*Yersinia pseudotuberculosis*	+	+	+	-
Number of riboswitches		31	50	59	28

**Table 2:** Validated criteria for the suitability of glmS, FMN, TPP, and SAM-I riboswitches for antibacterial drug targets. Riboswitches are widespread in bacterial species. The table presents data on the distribution of four of the most widely represented riboswitches in different bacterial species and among bacteria that infect humans. It also presents the criteria based on which we conclude about the possibility of each being used as a potential target for a new antisense oligonucleotide drug candidate. The ‘most suitable’ riboswitches (+++) control essential metabolites without alternative biosynthetic pathways and transport. The ‘very suitable’ riboswitches (++) control critical metabolites’ biosynthesis and transport. Suitable riboswitches are marked with +. The symbol ‘✔’ means the condition is fulfilled in all instances, ‘✔/-’ means the condition is fulfilled in some instances only, and ‘-’ means the condition is not.

Riboswitch’s Criteria	glmS Riboswitch	FMN Riboswitch	TPP Riboswitch	SAM-I Riboswitch
Number of bacterial species	912	2403	5624	2598
Number of human bacterial pathogens	31	50	59	28
Riboswitch-controlled biosynthetic pathway	✔	✔	✔	✔
Transporter protein for essential metabolite	-	✔	✔/-	✔
Without alternative biosynthetic pathways not under riboswitch control	-	✔	✔	✔
Suitability for drug targeting	+	+++	++/+++	+++

**Table 3:** Chimeric ASOs are designed to target four different bacterial riboswitches. The ASOs consist of a reverse complementary motif specifically sensed by its target. Index ‘_1_’ represents the 2′-alkyl modifications of the ribose, while index ‘_2_’ represents the phosphorothioate (PS) linkage. pVEC is attached to the 5′-end of the structure, marked in blue on the table.

ASO Name	pVEC_ASO Sequence 5′-3′	Nucleotides	ASO Target
pVEC_FMN_ASO_1	pVEC-T_1_T_1_C_1_T_2_C_2_C_2_C_2_A_2_T_2_C_2_C_2_A_2_G_2_A_1_C_1_T_1_	16nt	The aptamer of the FMN riboswitch
pVEC_FMN_ASO_2	pVEC-A_1_C_1_C_1_T_2_C_2_C_2_T_2_A_2_C_2_T_2_A_2_T_2_C_2_A_1_C_1_T_1_	16nt	Negative control for FMN riboswitch with 8 mismatches
pVEC_TPP_ ASO_1	pVEC-C_1_A_1_A_1_T_2_C_2_C_2_C_2_T_2_A_2_C_1_G_1_C_1_	12nt	The aptamer of the TPP riboswitch
pVEC_glmS_ASO_1	pVEC-C_1_T_1_T_1_T_2_A_2_A_2_C_2_T_2_G_2_T_2_A_2_C_2_T_2_G_1_C_1_C_1_	16nt	glmS riboswitch mRNA
pVEC_glmS_ASO_2	pVEC-T_1_G_1_C_1_T_2_T_2_C_2_T_2_T_2_C_2_G_2_T_1_A_1_T_1_	13nt	nagA mRNA
pVEC_SAM-I_ ASO_1	pVEC-T_1_C_1_C_1_C_2_T_2_C_2_C_2_A_2_C_2_C_2_A_2_C_1_T_1_C_1_	14nt	The aptamer of the SAM-I riboswitch

**Table 4:** Antimicrobial compounds targeting riboswitches.

Riboswitch Target	Name of the Antimicrobial Compound	Targeted Bacteria	References
**FMN**	pVEC_FMN_ASO_1	*E. coli* *L. monocytogenes* *S. aureus*	[13,19]
	Roseoflavin	*B. subtilis* *E.faecalis* *L. monocytogenes* *S. pyogenes*	[39,77,78]
	Ribocil	*E. coli*	[39,79]
	Ribocil-C	*E. coli* *S. aureus*	[79,80]
	Ribocil-C-PA	*K. pneumoniae*	[39,81]
	SFDQD	*B. suntilis* *Cl. difficile*	[39,82,83]
	10-(2,2-dihydroxylethy l)-7,8-di-methylisoalloxazine (5a)	*M. tubervulosis*	[6,81]
**glmS**	carba-α-D-glucosamine	*S. aureus*	[39,84]
	carba-α-D-glucosamine-6-phosphate	*S. aureus*	[39,84]
	fluoro- carba-α-D-glucosamine-6-phosphate	*B. subtilis* *S. aureus*	[39,85]
	pVEC_glms_ASO_1	*E. coli* *L. monocytogenes* *S. aureus*	[13,17]
**Guanine**	PC1	*S. aureus* *Cl. Difficile, MDR*	[39,86,87,88]
**nagA**	pVEC_glms_ASO_2	*S. aureus*	[13,17]
**SAM-I**	pVEC_SAM-I_ASO_1	*L. monocytogenes* *S. aureus*	[13,18]
**TPP**	Neomycin B	*B. subtilis* *S. aureus*	[89,90]
	PKZ18	*B. subtilis* *S. aureus*	[91]
	PKZ18-22	*B. subtilis* *S. aureus, MRSA*	[92,93]
	pVEC_TPP_ASO_1	*B. subtilis* *L. monocytogenes*	[13,14]
	Pyrithiamine	*B. subtilis*	[39,94]

## Data Availability

Not applicable.
